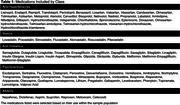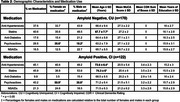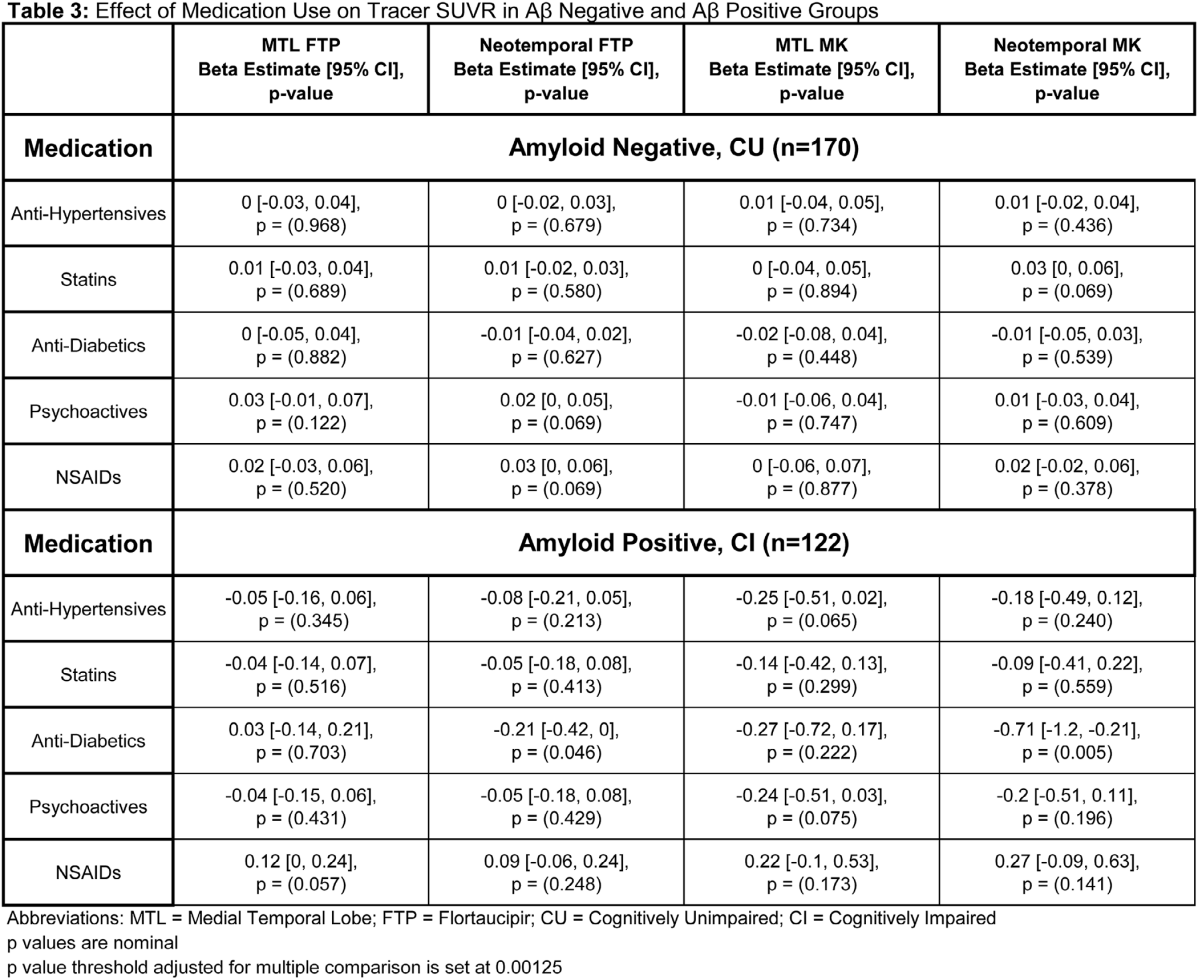# Impact of Medication Use on [18F]MK6240 and [18F]Flortaucipir Uptake in Alzheimer's disease

**DOI:** 10.1002/alz70862_110066

**Published:** 2025-12-23

**Authors:** Rayan Mroué, Pamela C.L. Ferreira, Guilherme Povala, Bruna Bellaver, Guilherme Bauer‐Negrini, Firoza Z Lussier, Livia Amaral, Marina Scop Madeiros, Emma Ruppert, Andreia Silva da Rocha, Matheus Scarpatto Rodrigues, Markley Silva Oliveira, Carolina Soares, Douglas Teixeira Leffa, Pampa Saha, Cynthia Felix, Joseph C. Masdeu, David N. soleimani‐Meigooni, Juan Fortea, Val J Lowe, Hwamee Oh, Belen Pascual, Brian A. Gordon, Pedro Rosa‐Neto, Suzanne L. Baker, Tharick A Pascoal

**Affiliations:** ^1^ University of Pittsburgh, Pittsburgh, PA USA; ^2^ Houston Methodist Research Institute, Houston, TX USA; ^3^ University of California, San Francisco, San Francisco, CA USA; ^4^ Sant Pau Memory Unit, Hospital de la Santa Creu i Sant Pau, Biomedical Research Institute Sant Pau, Barcelona Spain; ^5^ Mayo Clinic, Rochester, MN USA; ^6^ Brown University, Providence, RI USA; ^7^ Washington University in St. Louis, School of Medicine, St. Louis, MO USA; ^8^ Translational Neuroimaging Laboratory, The McGill University Research Centre for Studies in Aging, Montréal, QC Canada; ^9^ Lawrence Berkeley National Laboratory, Berkeley, CA USA

## Abstract

**Background:**

Quantifying tau aggregates in the human brain can be achieved using Positron Emission Tomography (PET) techniques, which can potentially be affected by binding competition due to medication use. Patients with dementia often have high rates of comorbidities and polypharmacy. Therefore, this study aims to investigate the potential influence of multiple medications on the uptake of the tau tracers MK6240 (MK) and Flortaucipir (FTP).

**Method:**

Five classes of medications were evaluated: Anti‐Hypertensives, Statins, Anti‐Diabetics, Psychoactive drugs, and NSAIDs (Table 1). We included 292 individuals [170 cognitively unimpaired (CU) Aβ‐negative and 122 cognitively impaired (CI) Aβ‐positive] from the HEAD study (Table 2). We compared MK and FTP SUVR in the Medial Temporal Lobe (MTL) and Neotemporal Cortex (NTC) in individuals on and off medications. The linear regressions that tested associations were corrected for confounding factors, including age, sex, education, and MoCA score. Correction for multiple comparisons was applied using the Bonferroni method (adjusted *p*‐value at 0.00125).

**Result:**

Among CI Aβ‐positive individuals, Anti‐Diabetics were associated with lower SUVR in the NTC for both FTP and MK. However, these associations did not remain significant after correction for multiple comparisons. (Table 3).

**Conclusion:**

Our findings indicate that there are no significant associations between the use of the medications studied and MK or FTP uptake when accounting for covariates and applying multiple comparison corrections.